# Impacts of Maize Domestication and Breeding on Rhizosphere Microbial Community Recruitment from a Nutrient Depleted Agricultural Soil

**DOI:** 10.1038/s41598-019-52148-y

**Published:** 2019-10-30

**Authors:** Vanessa L. Brisson, Jennifer E. Schmidt, Trent R. Northen, John P. Vogel, Amélie C. M. Gaudin

**Affiliations:** 1 Lawrence Berkeley National Laboratory, Berkeley, CA USA; 2 The DOE Joint Genome Institute, Walnut Creek, CA USA; 3 Lawrence Livermore National Laboratory, Livermore, CA USA; 4 Department of Plant Sciences, University of California at Davis, Davis, CA USA; 5 Department of Plant and Microbial Biology, University of California Berkeley, Berkeley, CA USA

**Keywords:** Microbial ecology, Microbial ecology, Plant domestication

## Abstract

Maize domestication and breeding have resulted in drastic and well documented changes in aboveground traits, but belowground effects on root system functioning and rhizosphere microbial communities remain poorly understood, despite their critical importance for nutrient and water acquisition. We investigated the rhizosphere microbial community composition and structure of ten *Zea mays* accessions along an evolutionary transect (two teosinte, three inbred maize lines, and five modern maize hybrids) grown in nutrient depleted soil from a low input agricultural system. Microbial community analysis revealed significant differences in community composition between soil compartments (proximal vs. distal rhizosphere) and between plant genetic groups (teosinte, inbred, and modern hybrid). Only a small portion of the microbial community was differentially selected across plant genetic groups: 3.7% of prokaryotic community members and 4.9% of fungal community members were significantly associated with a specific plant genetic group. Indicator species analysis showed the greatest differentiation between modern hybrids and the other two plant genetic groups. Co-occurrence network analysis revealed that microbial co-occurrence patterns of the inbred maize lines’ rhizosphere were significantly more similar to those of the teosintes than to the modern hybrids. Our results suggest that advances in hybrid development significantly impacted rhizosphere microbial communities and network assembly.

## Introduction

Since its origin in the Balsas River valley 10,000 years ago, maize (*Zea mays*) has become one of the most important and widely grown crops in modern agriculture, with over 700 million metric tons now being produced globally per year^1^. Maize domestication (the transition from teosinte to open pollinated maize cultivars) and modern breeding (the development of modern hybrids from their inbred parental lines) have traditionally emphasized selection on aboveground traits such as apical dominance and greater harvest index and seed size^2–4^. These processes have also influenced belowground traits including root architecture in maize^5–7^ and root exudate composition in wheat^8^, which in turn may affect the establishment of rhizosphere microbial communities in ways that are currently not clearly understood. Given the critical role of microorganisms in nutrient cycling and acquisition, increased understanding of the interactions between plants and rhizosphere microorganisms is an important step toward developing high yielding maize crops that require less resources, and enabling more sustainable agricultural systems.

Rhizosphere microorganisms are critical for supporting crop growth, especially under suboptimal conditions. They have been associated with improved uptake of scarce or otherwise poorly available nutrients^9,10^. Examples include solubilization and uptake of insoluble forms of phosphate and nitrogen fixation^10,11^. Some rhizosphere microorganisms can also decrease plant disease by directly competing with pathogens, producing antibiotic or antifungal compounds, or modulating plant immune responses^12^. Increased tolerance to environmental stresses, such as metal toxicity, and salts and drought stresses, has also been shown to be associated with particular rhizosphere microorganisms^11,13,14^. Some microorganisms can also produce and/or interfere with plant hormones such as auxins and cytokinins and more directly impact growth^15^.

The genotype of the host plant helps determine the physical and chemical rhizosphere environment influencing recruitment and establishment of microbial communities. Several studies have revealed a significant effect of plant genotype on rhizosphere microbial communities and regulation of ecological services provided by these plant associated microorganisms. In two studies comparing a range of grass species, including some crop plants, differences in both root and rhizosphere microbial communities have been found to be correlated with host plant phylogenetic distance^16,17^. Even among closely related maize inbred lines, significant differences have been identified among the rhizosphere microbial community compositions of different genotypes^18–20^. Similar dependence on host genotype has been seen in the rhizosphere communities associated with a variety of other plant species including potatoes, sweet potatoes, and the model plant *Arabidopsis thaliana*^9,21–23^.

Modern maize has been bred primarily under optimal cultivation conditions, selecting for traits associated with high yields in agricultural systems with high nutrient inputs. Thus, compared to older cultivars and wild relatives, modern cultivars may have lost some ability to recruit microorganisms that can support growth in low input conditions due to a lack of selective pressure^2,3^. Several reports have shown that domestication and breeding have affected the interactions of different crops with specific beneficial microorganisms as well as rhizosphere microbial communities. For instance, decreased interactions with arbuscular mycorrhizal fungi have been found in modern cultivars of wheat and maize as compared to older cultivars^3,24,25^. Studies have also revealed a reduced diversity of symbiotic rhizobia associations of domesticated legumes as compared to wild legumes^26,27^. In a study of sunflower rhizosphere communities along a gradient from wild to domesticated varieties, plant genotype was found to significantly influence fungal, but not bacterial community composition^28^. In maize, a recent study of one landrace from Mexico indicated that nitrogen fixing microorganisms were present and active in arieal root mucilage produced by this landrace, but not by modern maize hybrids^29^. However, the impacts of domestication and breeding on maize plant associated microbial communities remain uncertain: while a recent study of modern hybrids identified differences in rhizosphere microbial community composition even within this closely related set of genotypes^30^, comparison of two modern maize varieties with teosinte indicated significant differences between the microbial community diversity of the teosinte and one of the modern varieties, but not the other^31^.

Examination of microbial correlation networks is emerging as an important step to gain a deeper understanding of rhizosphere microbial communities and their ecological interactions^32^. These analyses can provide insights into the overall structure of the community and identify potential hub or keystone species based on their centrality and connectedness in the network structure^33,34^. A recent study of rhizosphere communities of wild oats (*Avena fatua*) identified a more complex and modular network structure in the rhizosphere as compared to bulk soil, and also showed that the complexity of the network increased over the course of plant development^33^. Coupling characterization of rhizosphere microorganisms with correlation network analysis could help clarify the ecological significance of small changes in community composition and recruitment, and provide further insight into plant regulation of beneficial interactions.

In this study, we investigate the possible impacts of domestication and modern breeding on rhizosphere microbial community recruitment from a low input agricultural soil depleted in most nutrients, an environment in which recruitment of beneficial microorganisms is important to sustain crop production. We analyze the rhizosphere microbial communities associated with 10 *Zea mays* accessions representing three plant genetic groups (teosinte, inbred maize lines, and modern maize hybrids) along an evolutionary transect of maize domestication and breeding. We examine impacts on both community composition and microbial correlation network structure in order to improve our understanding of the extent to which plant-microbial interactions may have changed or been lost during maize evolution. Our aim was to determine whether there were any identifiable shifts in microbial community composition and network structure associated with the transitions of domestication and breeding represented by these plant groups. Understanding these changes has the potential to open new avenues to identify and promote beneficial interactions with the goal to ecologically intensify agroecosystems and enable more sustainable, lower input agriculture. It will also facilitate further studies to ultimately incorporate plant-microbial interactions into plant breeding programs.

## Results

### The rhizosphere environment and plant genetic group impact microbial community composition

Across all plant groups, soil compartment impacted both α- and β-diversity, while effects of plant genetic group were only seen in β-diversity. After rarefication and removal of low prevalence amplicon sequence variants (ASVs) and chloroplast and mitochondrial ASVs, 2790 prokaryotic ASVs were identified (2771 bacterial and 19 archaeal ASVs), and 189 fungal ASVs were identified.

**Figure 1:**
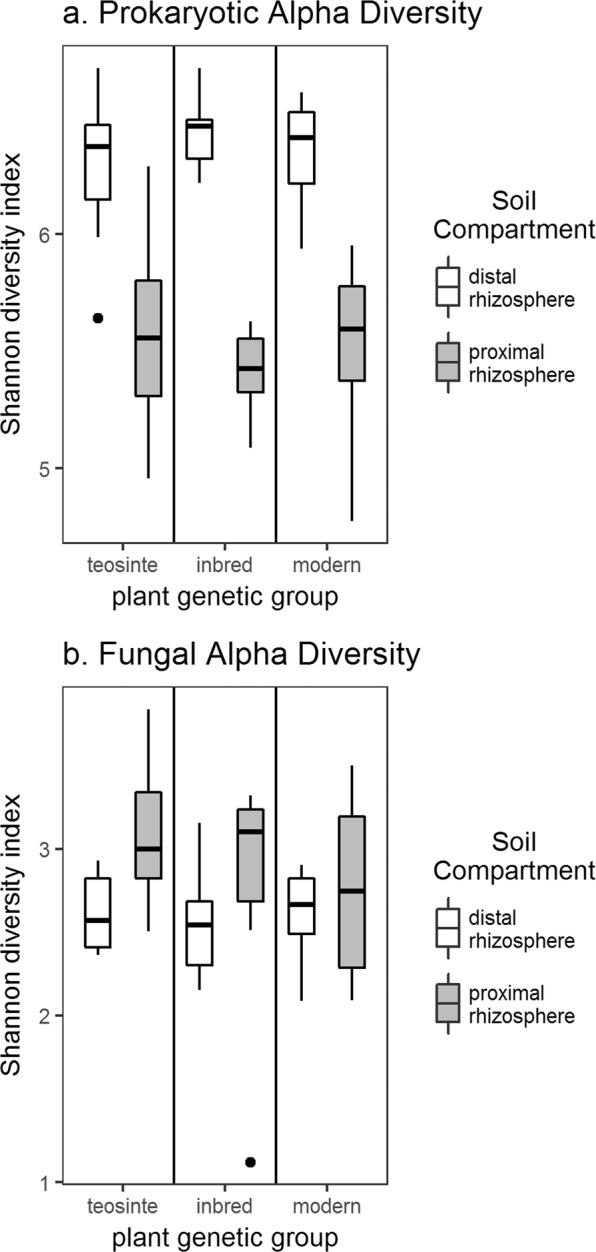
Microbial community α-diversity (Shannon diversity index) of proximal and distal rhizosphere samples from different plant genetic groups based on (**a**) prokaryotic 16S-V4 sequences, and (**b**) fungal ITS2 sequences.

Prokaryotic community α-diversity, as measured by the Shannon diversity index, was consistently greater in the distal rhizosphere than in the proximal rhizosphere soil (Fig. 1a). Two way type III analysis of variance (ANOVA) indicted that the effect of soil compartment was significant (p < 0.001), while the effects of plant group and interaction effects were not (Supplementary Table S1). This trend was reversed in the fungal community, where α-diversity was higher in proximal rhizosphere soil than in the distal rhizosphere (p = 0.018) (Supplementary Table S2). The effect was more pronounced for the teosinte and inbred plant genetic groups than for modern hybrids (Fig. 1b). However, no significant differences in α-diversity were detected for different plant genetic groups or for interactions between plant genetic groups and soil compartments. This trend of lower prokaryotic diversity and higher fungal diversity in the proximal rhizosphere was further supported by an analysis of six additional α-diversity measures: observed number of ASV’s, Chao1 richness, abundance based coverage estimator index, Shannon diversity index, Simpson index, inverse Simpson index, and Fisher’s alpha (Supplementary Fig. S1).

**Figure 2:**
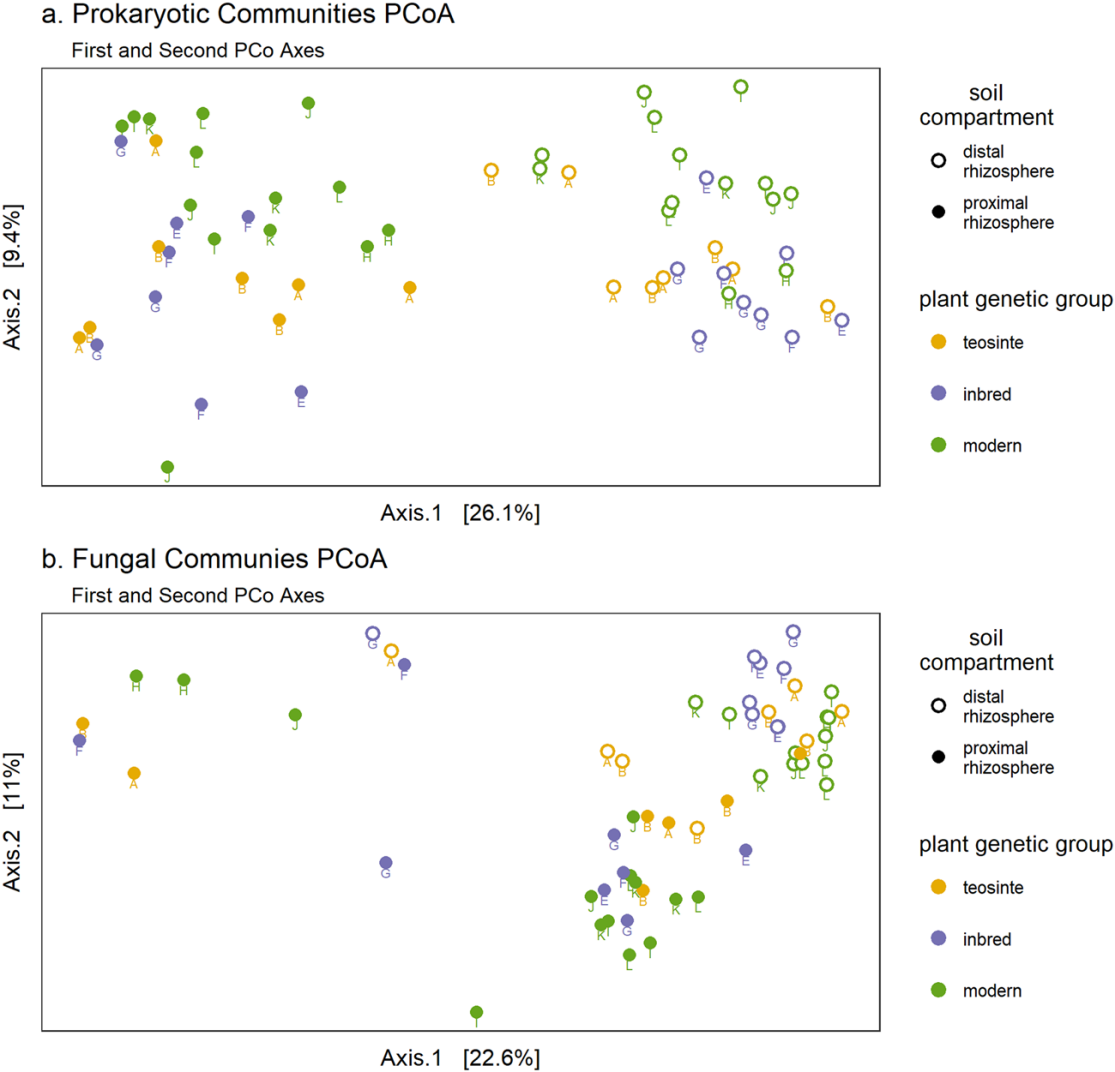
PCoA plots based on Bray-Curtis distances between samples for (**a**) prokaryotic 16S-V4 sequences, and (**b**) fungal ITS2 sequences. Filled and open circles indicate proximal and distal rhizosphere samples respectively. Colors indicate different plant genetic groups. Letters indicate plant genotype as listed in Table 1.

Prokaryotic community β-diversity, as measured using Bray-Curtis distances, was influenced by both soil compartment and plant genetic group. Principal coordinate analysis (PCoA) showed clustering of samples by soil compartment along the first principal coordinate axis and clustering by plant genetic group along the second principal coordinate axis (Fig. 2a). Two way permutational analysis of variance (PERMANOVA) indicated that both soil compartment and genotype effects were significant, but their interaction effect was not (Supplementary Table S3). Soil compartment accounted for 23% of the variance (p = 0.001) and plant genetic group accounted for 7.7% of the variance (p = 0.002). The two way PERMANOVA of fungal community β-diversity indicated a significant effect of soil compartment (12% of variance, p = 0.001), whereas the effect of plant genetic group was weaker, although still statistically significant (5.4% of variance, p = 0.019), and the interaction was not significant (Supplementary Table S4). PCoA of the fungal microbial community revealed that samples also clustered by soil compartment along a combination of the first two principal coordinates (Fig. 2b), whereas clustering by plant genetic group was not visibly evident, consistent with the small effect size in the PERMANOVA analysis.

**Figure 3:**
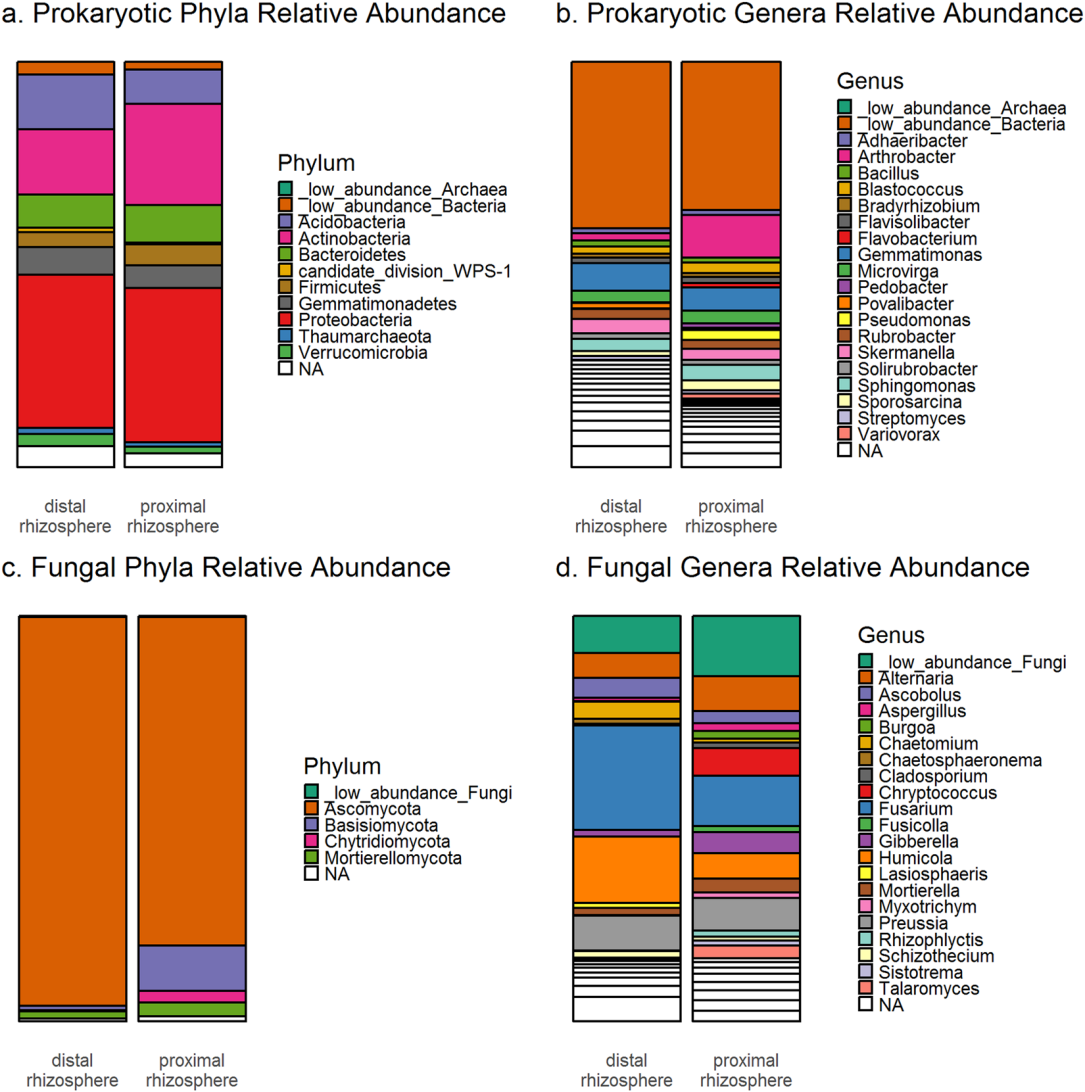
Relative abundance of different taxonomic groups represented within the (**a**,**b**) prokaryotic 16S-V4 sequences, and (**c**,**d**) fungal ITS2 sequences. Taxa are clustered at the (**a**,**c**) phylum and (**b**,**d**) genus levels. Low abundance taxa, defined as those below 1% abundance in all sample groups, are grouped together. Identifications are based on the RDP database for prokaryotes and the UNITE ITS database for fungi.

The relative abundance of different taxonomic groups shifted between the proximal and distal rhizosphere samples. Most notably in the prokaryotic community, there was a shift in the phylum Actinobacteria to a higher abundance in the proximal rhizosphere (Fig. 3a). That shift was dominated by representatives of the family Micrococcaceae, which included the genus *Arthrobacter* (Fig. 3b). This effect is consistent across all proximal and distal rhizosphere samples (Supplementary Fig. S2). This finding was supported by a differential abundance analysis, which indicated that four Micrococcaceae ASVs (16s_ASV_1, 16S_ASV_23, 16S_ASV_45, and 16S_ASV_155) were enriched in the proximal compared to distal rhizosphere samples (log2 fold changes = 1.2, 2.6, 0.8, and 1.1 respectively, p_adjusted_ < 0.001 for all three ASVs).Differential abundance analysis also revealed enrichment in the proximal rhizosphere for the genera *Pseudomonas*, *Variovorax*, *Sporosarcina* (alternatively identified as *Paenisporosarchina* based on the Silva database), and *Flavobacterium*, each represented by one to three ASVs (p_adjusted_ < 0.001). Proximity to the root also influenced fungal communities (Fig. 3c,d). While the distal rhizosphere fungal community was heavily dominated by the Ascomycota phylum, the proximal rhizosphere had significantly increased abundances of Basidiomycota and Chytridiomycota (log2 fold changes = 4.2 and 4.5, p_adjusted_ < 0.001) (Fig. 3c,d, Supplementary Fig. S3).

### Plant genetic groups selectively recruit a subset of low abundance ASVs

Differential abundance and indicator species analyses identified ASVs that were highly associated with plant genetic groups. These analyses were performed on proximal rhizosphere samples to focus on the region most directly under the influence of plant roots.

**Figure 4:**
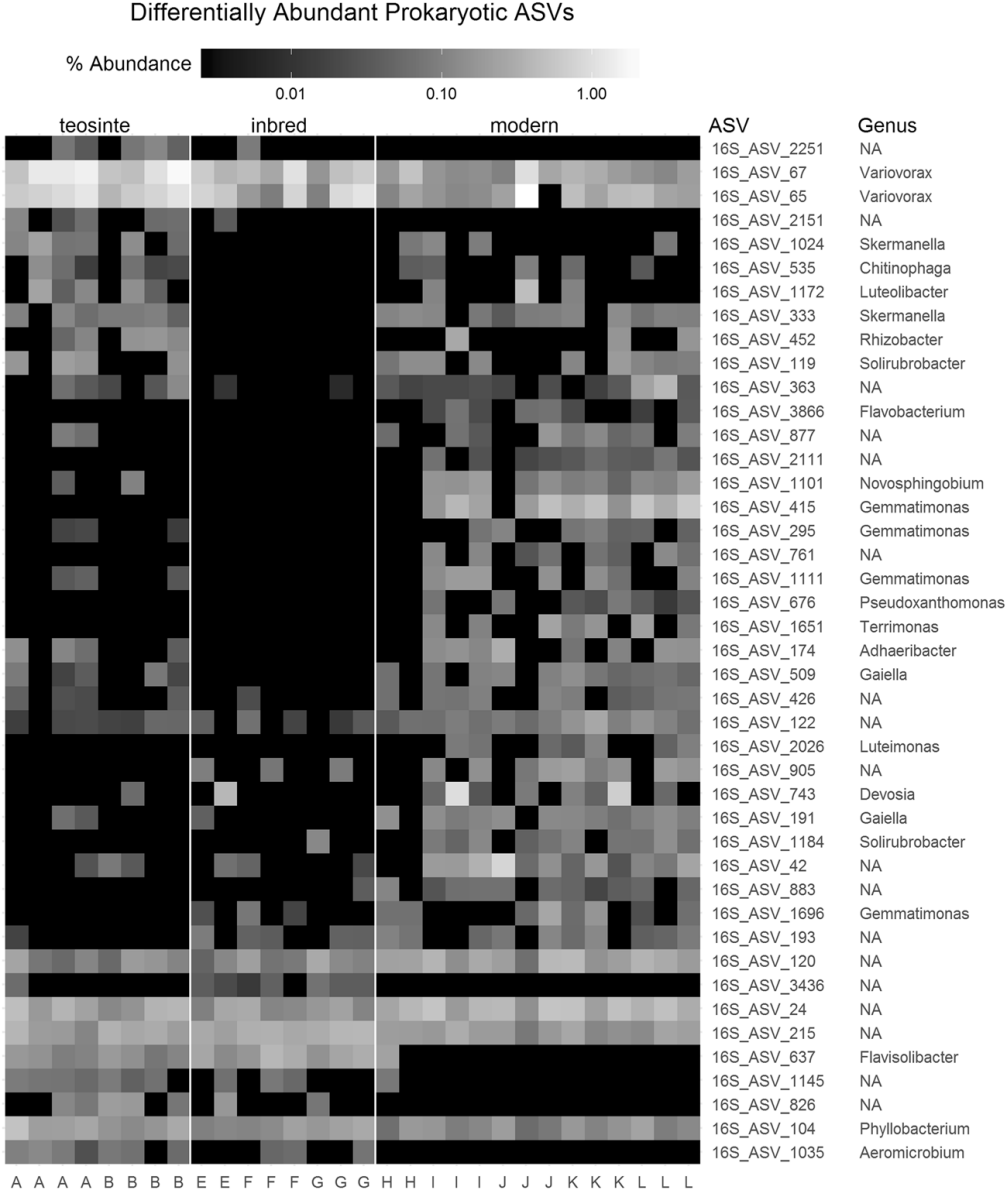
Differentially abundant prokaryotic ASVs detected by DESeq2 at a significance level of p_adjusted_ < 0.05. All ASVs that were differentially abundant between any pair of plant genetic groups are included. Rows represent individual ASVs and columns represent individual samples. ASVs are ordered by hierarchical clustering based on their relative abundance patterns across all samples. Samples are grouped by plant genetic group as shown at the top. Within plant genetic groups, samples are grouped by plant accession, indicated by letters at the bottom corresponding to accession IDs listed in Table 1. Brightness indicates ASV relative abundance on a logarithmic scale.

Forty-three prokaryotic ASVs (3.7% of all prokaryotic ASVs identified in the proximal rhizosphere samples) were differentially abundant between at least two of the three plant genetic groups (Fig. 4). These were low relative abundance organisms, with only two ASVs having abundances greater than 1% in any sample. These were 16S_ASV 65 and 16S_ASV_67, with maximum relative abundances of 2.0% and 1.7% respectively. Both of these ASVs were identified as belonging to the genus *Variovorax*, and had a higher relative abundance in teosinte proximal rhizosphere samples than those obtained from modern hybrids. Additionally, five differentially abundant ASVs (16S_ASV_676, 16S_ASV_1145, 16S_ASV_2111, 16S_ASV_3436, and 16S_ASV_3866) were only present at abundances below 0.1% in all samples (0.080%, 0.086%, 0.075%, 0.066%, and 0.069% maximum relative abundance respectively). Fifteen of the differentially abundant ASVs could not be identified at the genus level using either the RDP or Silva databases.

Indicator species analysis identified 40 prokaryotic ASVs (3.4%) as indicators of plant genetic groups or pairs of plant genetic groups (Table 1, Supplementary Fig. S4). The greatest number of indicator ASVs (12) were associated with both teosinte and inbred groups, meaning that they were generally present in the rhizospheres of both of these groups, but absent from the rhizospheres of the modern hybrid accessions. Additionally, 11 ASVs were indicator species for the modern hybrid plant genetic group and 10 ASVs were indicators for teosinte. There was significant overlap between the indicator species analysis and the differential abundance analysis, with 18 ASVs (45% of indicator species) being identified as being associated with particular plant groups by both analyses (Table 1, bold).

**Table 1:** Indicator Species Analysis. Indicator ASVs associated with each plant group and with pairs of plant groups. ASVs also identified in the differential abundance analysis are indicated in **bold**. ASV identifications are based the RDP database. Identifications based on the Silva database are provided in parenthases wherever they differ from the RDP identifications.

Plant Group	ASV	Kingdom	Phylum	Genus
teosinte	16S_ASV_487	Bacteria	NA (Chloroflexi)	NA
	**16S_ASV_1024**	**Bacteria**	**α-Proteobacteria**	***Skermanella***
	**16S_ASV_1035**	**Bacteria**	**Actinobacteria**	***Aeromicrobium***
	**16S_ASV_1145**	**Bacteria**	**Verrucomicrobia**	**NA (** ***Chithonibacter*** **)**
	16S_ASV_1279	Bacteria	NA (Gemmatimonadetes)	NA
	16S_ASV_1416	Bacteria	α-Proteobacteria	NA
	**16S_ASV_2151**	**Bacteria**	**Chloroflexi**	**NA**
	16S_ASV_2781	Bacteria	Bacteroidetes	*Flavobacterium*
	16S_ASV_4013	Bacteria	α-Proteobacteria	*Sandaracinobacter* (NA)
	16S_ASV_4121	Bacteria	γ-Proteobacteria	NA
inbred	**16S_ASV_1500**	**Bacteria**	**Acidobacteria**	NA
	16S_**ASV_1914**	**Bacteria**	**Acidobacteria**	NA (*Candidatus Solibacter*)
	**16S_ASV_3436**	**Bacteria**	**δ-Proteobacteria**	NA
modern	**16S_ASV_191**	**Bacteria**	**Actinobacteria**	***Gaiella*** **(NA)**
	**16S_ASV_415**	**Bacteria**	**Gemmatimonadetes**	***Gemmatimonas***
	**16S_ASV_676**	**Bacteria**	**γ-Proteobacteria**	***Pseudoxanthomonas***
	**16S_ASV_761**	**Archaea**	**Thaumarchaeota**	NA
	**16S_ASV_883**	**Bacteria**	**α-Proteobacteria**	NA
	**16S_ASV_1101**	**Bacteria**	**α-Proteobacteria**	***Novosphingobium***
	**16S_ASV_1184**	**Bacteria**	**Actinobacteria**	***Solirubrobacter*** **(NA)**
	16S_ASV_1650	Bacteria	α-Proteobacteria	*Sphingobium*(NA)
	16S_ASV_1722	Bacteria	Bacteroidetes	*Flavisolibacter*
	**16S_ASV_2111**	**Bacteria**	**Actinobacteria**	NA
	**16S_ASV_3866**	**Bacteria**	**Bacteroidetes**	***Flavobacterium***
teosinte & inbred	16S_ASV_162	Bacteria	α-Proteobacteria	*Bradyrhizobium* (NA)
	16S_ASV_297	Bacteria	Gemmatimonadetes	*Gemmatimonas*
	16S_ASV_352	Bacteria	Gemmatimonadetes	*Gemmatimonas*
	16S_ASV_485	Bacteria	γ-Proteobacteria	*Luteimonas*
	16S_ASV_512	Bacteria	Actinobacteria	*Pseudonocardia*
	**16S_ASV_637**	**Bacteria**	**Bacteroidetes**	***Flavisolibacter***
	16S_ASV_774	Bacteria	Gemmatimonadetes	*Gemmatimonas*
	16S_ASV_827	Bacteria	α-Proteobacteria	*Sphingomonas*
	16S_ASV_901	Bacteria	Chloroflexi	NA
	16S_ASV_1301	Bacteria	Verrucomicrobia	*Roseimicrobium*
	16S_ASV_1603	Bacteria	NA	NA
	16S_ASV_2203	Bacteria	Acidobacteria (Chloroflexi)	NA
teosinte & modern	**16S_ASV_333**	**Bacteria**	**α-Proteobacteria**	***Skermanella***
	**16S_ASV_509**	**Bacteria**	**Actinobacteria**	***Gaiella (NA)***
inbred & modern	16S_ASV_364	Bacteria	Gemmatimonadetes	*Gemmatimonas*
	**16S_ASV_193**	**Bacteria**	**Acidobacteria**	**NA**

Six fungal ASVs (ITS_ASV_15, ITS_ASV_83, ITS_ASV_163, ITS_ASV_196, ITS_ASV_199, and ITS_ASV_270) were identified as differentially abundant between plant genetic groups (Supplementary Fig. S5), with maximum relative abundances within the fungal community ranging from 0.65% to 4.2%. These included six Ascomycota and one Basidiomycota (ITS_ASV_196). No fungal ASVs were identified as indicator species for any plant genetic group. This is consistent with the weaker effect of plant genetic group on fungal community composition reported above (Fig. 2b).

### Microbial co-occurrence networks are more different in modern hybrids

**Figure 5:**
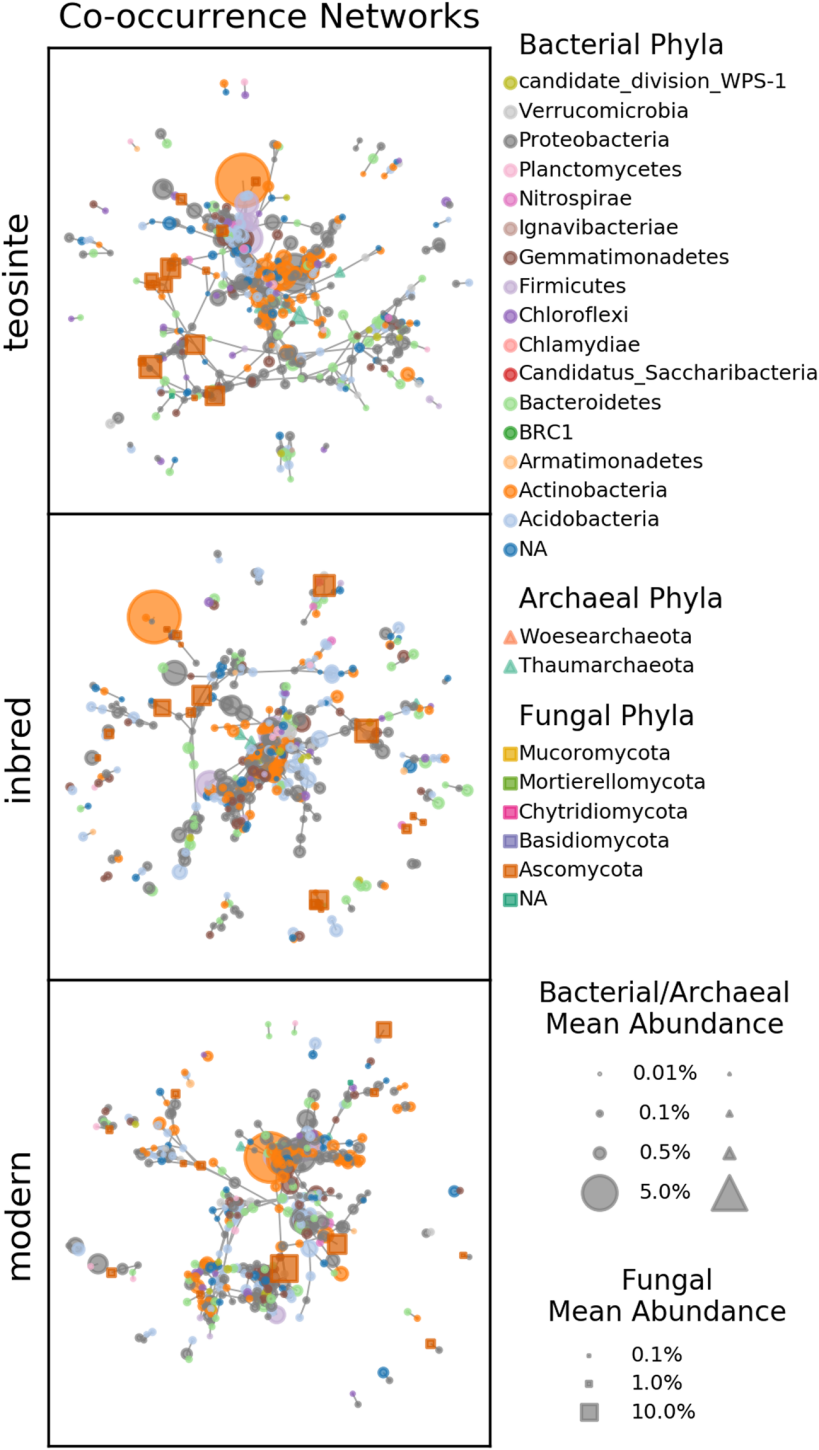
Microbial co-occurrence networks constructed at a minimum correlation level of ρ_min_ = 0.85. Each node in a network represents an ASV, and each edge represents a significant positive correlation. Node shapes indicate the kingdom, colors indicate the ASV phylum, and sizes represent the ASV’s mean relative abundance in samples contributing to that network. Only ASVs having at least one significant correlation are included in the network.

Microbial co-occurrence networks were constructed for each of the three plant genetic groups. Network properties were evaluated for a range of minimum correlation coefficients ρ_min_ between 0.5 and 0.95. Properties of the networks are summarized in Supplementary Fig. S6. With increasing ρ_min_, the number of nodes, number of edges, and mean degree decreased, as could be expected with increased stringency of the correlation detection. The teosinte networks consistently had the highest number of nodes of the three plant genetic groups across all ρ_min_ levels (Supplementary Fig. S6a), indicating that more ASVs in the teosinte rhizosphere data were positively correlated with at least one other ASV. The mean degree (number of connections per ASV) was also consistently highest for the teosinte network, indicating a more densely connected network (Supplementary Fig. S6c). This high density of connections is also evident in network visualizations at ρ_min_ = 0.85 (Fig. 5).

**Figure 6:**
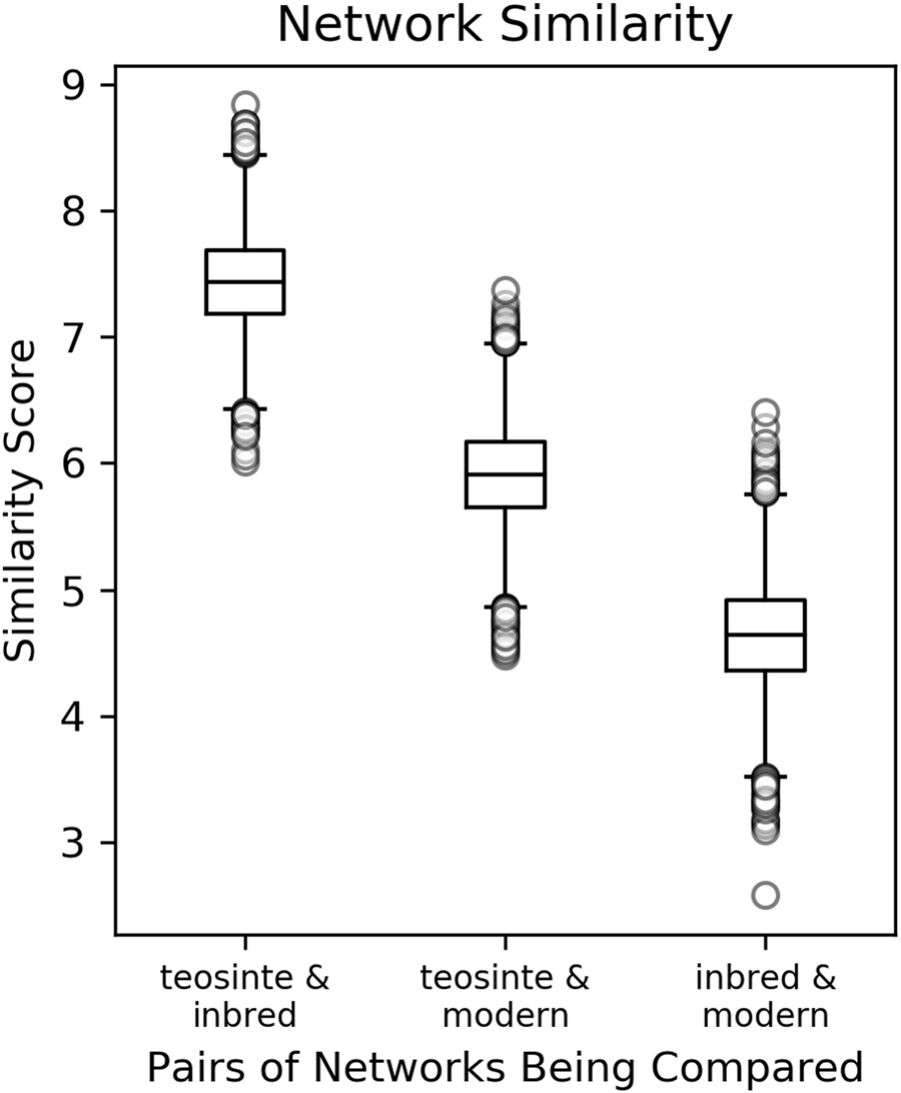
Similarity of pairs of co-occurrence networks. The similarity score is the percent reduction (compared to randomly resampled correlation matrices) in the matrix norm of the difference between the two correlation matrices being compared (Eq. 1). Data represent 10,000 resamplings of the data to calculate similarity scores.

The microbial correlation matrices used to construct the networks were compared to assess similarity between co-occurrence patterns among plant genetic groups. We observed the greatest similarity between the inbred and teosinte networks, and the lowest similarity between the inbred and modern hybrid networks (Fig. 6). The similarity scores for all three pairs of networks were significantly different from each other (p ≤ 0.012).

All putative keystone taxa identified in the co-occurrence networks were Bacteria, and all but one belonged to one of the six most dominant phyla in the rhizosphere microbial communities: Acidobacteria, Actinobacteria, Bacteroidetes, Firmicutes, Gemmatimonadetes, and Proteobacteria (Supplementary Table S5, Fig. 3). The one other putative keystone taxon could not be identified at the phylum level based on the RDP database, but was identified as Tectomicrobia based on the Silva database. Only 2 of the 50 putative keystone taxa had mean relative abundances above 1%, while 10 had mean relative abundances of less than 0.1%.

The majority of identified putative keystone taxa were distinct between the networks. Only one, a *Skermanella* species, was a shared putative keystone for the networks of two plant genetic groups, teosinte and modern hybrids. Five of the putative keystone taxa were also identified as differentially abundant or indicator species, having a higher association with the same plant genetic group for which they were a keystone (Table 1, Fig. 4, Supplementary Table S5). These included three for the teosinte network (a *Gemmatimonas*, a *Pseudonicardia*, and a *Flavisolibacter*), and two for the modern hybrid (a *Solirubacter* and a *Gemmatimonas*).

## Discussion

We explored the unintended impact of aboveground selection for high yields during maize domestication and modern breeding on rhizosphere microbial community recruitment and assembly from a nutrient depleted agricultural soil. The importance of rhizosphere microorganisms in supporting plant growth under suboptimal growth conditions has been well documented^9–11,13,14^. Thus, the ability of plants to recruit potentially beneficial microorganisms to the rhizosphere when grown in a nutrient poor soil is of particular relevance to potential changes in recruitment ability over the course of domestication and breeding.

We found greater similarity between the rhizosphere microbial communities associated with the inbred maize lines and those of teosinte as compared to modern hybrids, suggesting a greater impact of modern breeding than initial domestication on these microbial communities. Specifically, the analysis of rhizosphere community composition showed significant differences between the plant genetic groups, with the inbred and modern hybrid groups appearing most distinct and the teosinte being intermediate (Fig. 2). At the individual ASV level, both the differential abundance and indicator species analyses showed greater overlap between the teosinte and inbred plant groups (Fig. 4, Supplementary Fig. S4). These findings were reinforced by our assessment of the similarity of co-occurrence network structures and underlying correlation matrices, which revealed the greatest similarity between the inbred and teosinte networks and the least similarity between the inbred and modern hybrid networks (Fig. 6). Other studies have found some correlation between phylogenetic distance and rhizosphere microbial community dissimilarity in maize and other grasses^16,17^ which would predict that the rhizosphere communities of the inbred lines should have greater similarity to modern hybrids than teosinte. One possible explanation for this apparent discrepancy is that heterosis and breeding under high nutrient inputs following the Green Revolution dramatically altered plant rhizosphere recruitment traits. In a recent study of twelve modern maize hybrids, rhizosphere community composition was not correlated with chronological release date, suggesting that there was not a consistent trajectory of microbial community composition over the range of modern hybrids investigated^30^. Our results reinforce those findings of a non-linear trajectory for microbial community composition, and expand them to the larger context of maize domestication and breeding leading up to modern hybrids.

The experimental context is important for understanding the implications of our results and possible drivers of the trends observed. In the selection of plant accessions, we endeavored to cover an evolutionary transect of domestication and breeding from teosinte through modern maize hybrids (Table 2), and included at least two accessions from each plant group in order to reduce potential biases associated with individual accessions. However, this panel does not cover the genetic diversity of *Zea maize*, and more variation should be examined to conclude on the effects of directed selection aboveground on microbial communities belowground. Instead, this work should be placed in the context of other work, bridging the space between studies focusing either on a phylogenetic range of more distantly related grasses^16,17^ or on greater genetic diversity within maize hybrids^30^.

**Table 2:** Plant Accessions.

Plant Group	Accession ID	Source	Description	ID for this study
Teosinte	PI 566688	Mexico	Pre-domestication, wild ancestor	A
	PI 566691	Mexico	Pre-domestication, wild ancestor	B
Inbred	B73 (PI 550473)	USA	Parents of modern germplasm	E
	Mo17 (PI 558532)	USA	Parents of modern germplasm	F
	OH43 (Ames 19288)	USA	Parents of modern germplasm	G
Modern Hybrid	322HYB	USA	Released 1936; double cross hybrid	H
	354HYB	USA	Released 1953; double cross hybrid	I
	3382	USA	Released 1976; single cross hybrid	J
	3489	USA	Released 1994; single cross hybrid	K
	DeKalb2015	USA	New release (2015–16)	L

Trends in the diversity and composition of the rhizosphere microbial communities in this study were consistent with expectations. Various studies have found that these communities are often dominated by the same phyla with the greatest relative abundances in this study: Acidobacteria, Actinobacteria, Bacteroidetes, Firmicutes, Gemmatimonadetes, and Proteobacteria (Fig. 3a, Supplementary Fig. S2)^23,35,36^. In addition, the α-diversity of the prokaryotic microbial community in the proximal rhizosphere was significantly lower than that of the distal rhizosphere. Other studies that have also reported lower α-diversity closer to the root, where more selective influence is expected from the plant^19,37^. We also identified significant differences in microbial community composition between soil compartment (proximal and distal rhizosphere) and plant genetic group (teosinte, inbred, and modern hybrid). Plant genetic group has a relatively small impact compared to the overall influence of the root, which is consistent with previous studies which have identified larger effects of sample type than plant genotype on β-diversity^19,36,37^. In contrast to the prokaryotic community, the fungal community had greater α-diversity in the proximal rhizosphere as compared to the distal rhizosphere. Some studies have reported fungal diversity showing similar trends to prokaryotic diversity^38^. However, fungal communities are more seldom reported in rhizosphere microbial community studies. Astudy of different *Agave* species found that fungal diversity was higher in the rhizosphere than in the root zone (analogous to proximal and distal rhizosphere in this study) for three of the four *Agave* species studied^37^ and more information and meaningful integration of fungal communities in rhizosphere studies are necessary.

Differentially abundant microorganisms and indicator species may be of interest for detailed examination of the mechanisms by which these plant genetic groups differentially attract potentially beneficial microorganisms, presumably through exudates^39^. In this study we used nutrient depleted soils to identify recruitment pf potentially adaptive taxa, especially in Teosinte. We find that the differentially abundant taxa with the highest relative abundance were two ASVs identified as *Variovorax*, which reached up to 2% abundance. Although present in almost all rhizosphere samples, these ASVs showed higher relative abundance in the teosinte plant genetic group (Fig. 4). *Variovorax* species have been isolated from rhizosphere samples of various plants, and some strains have been shown to have plant growth promoting properties, including growth promotion in maize, making them promising targets for further study^40,41^.

Five ASVs identified as differentially abundant or as indicator taxa are also putative keystone species in the co-occurrence network analysis and likely play important roles in these communities. These include representatives of four genera: *Gemmatimonas*, *Pseudonocardia*, *Flavisolibacter*, and *Solirubrobacter*. Although the roles of these ASVs cannot be determined from the data in this study, other studies of members of these genera may provide some insight into their importance. For instance *Flavisolibacter* is known to vary in abundance over time during maize growth^42^. This indicates a responsiveness to variation in the maize rhizosphere that is further supported by our observed responsiveness to differences between the related plant genetic groups. Future research should test whether network reorganizations observed across the different genetic groups result in different functional properties such as changes in susceptibility to pathogen invasion or greater benefits to the plant host, two functional outcomes that have been linked in theory to network properties^43^.

This study focused on microbial communities in the rhizosphere because this zone represents the interface between the plant and the soil and is a critical location for nutrient cycling and plant-soil-microbe interactions of relevance for agricultural production and sustainability. Although the microbial communities of the root endosphere are also important for supporting plant growth^44,45^ and, in some cases, correlate with plant host phylogeny^17,46^, these communities were outside the scope of this study. Nevertheless, further studies investigating the interplay between endosphere and rhizosphere could provide additional insights into the significance of these communities for maize growth.

The results of our study suggest that over the course of maize evolution there have been changes in rhizosphere microbial community recruitment, but that the changes associated with modern breeding under optimal high input conditions may have been greater than those associated with domestication. The microbial community composition, as well as the microbial correlation network structure, of the inbred maize lines were found to be more similar to those of the teosinte lines, and less similar to the modern hybrids. The consequences of decoupling rhizosphere processes from plant selection for maize adaptation and production in low input systems remain unclear, but this long history of co-evolution likely provides a useful roadmap for measuring and managing beneficial plant-microbe interactions that are key components of sustainable maize production.

## Methods

### Plant accessions representing wild relatives, ancestral maize, and modern maize hybrids

Ten *Zea mays* accessions were selected to represent three plant genetic groups at three time point along an evolutionary transect of maize domestication and breeding: two teosinte accessions (*Zea mays* ssp. *parviglumis*, wild relative from which maize was domesticated), three inbred maize accessions that were the parents of modern hybrids, and five modern hybrids (Table 2). Four of the hybrids were from the Pioneer ERA Panel, which contains well-studied, commercially successful hybrids ranging in release date from 1936 to 1994^47^, and one was a more recent release from 2015.

### Plant growth in nutrient depleted agricultural soil

Plants were grown in a greenhouse at the University of California, Davis in five gallon pots filled with field soil from the Century Experiment located at the Russell Ranch Sustainable Agriculture Facility at the University of California, Davis^48^. The soil was collected in August of 2017 from rain fed plots under a wheat/fallow rotation for 23 years without fertilization or supplemental irrigation, resulting in nutrient depleted soil conditions with low levels of nitrate and phosphorus (Supplementary Table S6). Top soil (0–20 cm) was collected across three replicate plots and thoroughly mixed to produce a homogenized soil. Seeds were surface sterilized in 5% sodium hypochlorite for one minute and pre-germinated in petri dishes for three days. One seed per pot was carefully transplanted at a depth of 2.5 cm. Four replicates per maize accession were grown for 8 weeks without supplemental lighting in a complete randomized block design. Plants were grown in 5 gallon (19 L) pots and watered daily using automatic drip irrigation without fertilization.

### Collection of proximal and distal rhizosphere soil samples

Separate proximal and distal rhizosphere samples were collected for microbial community analysis. Plants were root bound, and thus all soil to the depth of rooting was likely under the influence of the root. Therefore, we defined two separate zones: the proximal rhizosphere was defined as soil adhering to the roots, while distal rhizosphere was defined as soil to the depth of rooting that was not closely adhering to the root but that came from the same pot. Proximal rhizosphere soil samples were collected using a modified version of the protocol described by Barillot *et al*.^49^. Briefly, roots were gently shaken to remove non-adhering soil. For each plant, three 5 cm root segments collected 5 cm from the crown were placed in 50 mL centrifuge tubes with 10 mL of sterile solution containing 0.9% NaCl and 0.01% Tween. Samples were incubated in an orbital shaker for 90 minutes at 300 rpm to remove adhering soil. Following shaking, roots were removed, and samples were centrifuged at 14,000 rpm (39,400 g) for 10 minutes to pellet soil and cells. The supernatant was discarded and the centrifuged samples were frozen at −80 °C until DNA extraction.

### DNA extraction and 16S-V4 and ITS2 sequencing

DNA was extracted using the MoBio PowerSoil DNA Isolation Kit [MoBio Laboratories Inc., Carlsbad, CA, USA] according to the manufacturer’s instructions. Amplicon sequencing was performed at the DOE Joint Genome Institute. The prokaryotic 16S-V4 region was amplified using the forward primer 515F-Y (GTGYCAGCMGCCGCGGTAA)^50^ and reverse primer 806 R (GGACTACNVGGGTWTCTAAT)^51^. The fungal ITS2 region was amplified using the forward primer ITS9F (GAACGCAGCRAAIIGYGA) and reverse primer ITS4R (TCCTCCGCTTATTGATATGC)^52,53^. Libraries were prepared according to the DOE Joint Genome Institute’s iTag library construction protocol (http://1ofdmq2n8tc36m6i46scovo2e-wpengine.netdna-ssl.com/wp-content/uploads/2017/08/iTag-Sample-Preparation-for-Illumina-Sequencing-SOP-v1.0.pdf)and sequenced on an Illumina MiSeq sequencer in 2 × 300 run mode.

16S-V4 amplicons were sequenced from a total of 61 samples, and ITS2 amplicons were sequenced from 60 samples. The discrepancy between the number of plants originally planted and the number of samples sequenced was the result of sample loss to either pre-sampling plant death or poor yields in DNA extractions for some samples. For 16S-V4 sequencing data, all 61 samples (30 proximal and 31 distal rhizosphere) sequenced had greater than 10,000 reads, a threshold selected to remove low quality sequencing data. Data for all samples were rarefied to the minimum read total of 42,309 reads. For ITS2 sequencing data, 2 out of 60 samples sequenced had fewer than 10,000 reads and were removed from the analysis. Data for the remaining 58 samples (29 proximal and 29 distal rhizosphere) was rarefied to the minimum remaining read total of 14,767 reads.

### Microbial community analysis

Raw sequence reads were trimmed to remove primer sequences from both the 5’ and 3’ ends of reads using cutadapt version 1.14^54^. Trimmed reads were analyzed using the DADA2 package in R, following a combination of the standard and big data pipelines for paired reads^55^. Read quality profiles were visualized for forward reads from three randomly selected samples and for reverse reads from three different randomly selected samples. Based on those visualizations, reads were filtered and trimmed with the following parameters (16S-V4 sequences: truncLen = (160, 120), maxEE = (2, 5), and truncQ = 2; ITS2 sequences: no truncLen, maxEE = (2, 2), and truncQ = 11). Following trimming, error rates were estimated based on one million reads. Sequences were dereplicated and chimeras were removed using the “consensus” method. ASV taxonomy was initially assigned using the RDP database for 16S-V4 sequences^56^. Some evidence suggests that RDP has a lower error rate than other commonly used databases^57^. However, because the RDP database has not been updated since 2016, identifications based on the more recently updated Silva database were also determined and are included for comparison^58^. The UNITE ITS database was used to assign taxonomy to ITS2 ASVs^59^. Resulting ASV tables were further processed and analyzed with the phyloseq package in R^60^. 16S-V4 ASVs corresponding to chloroplast or mitochondrial sequences and ITS2 ASVs corresponding to non-fungal sequences were removed. Samples with fewer than 10,000 reads were discarded to remove low quality data, and data were rarified to the minimum sequencing depth of the remaining samples. Extremely low prevalence ASVs, defined as those present in fewer than six samples, were removed from downstream analyses.

α-diversity indices were calculated using the “estimate_richness” and “plot richness” functions in the phyloseq analysis package in R. A two way type III ANOVA was performed to investigate the significance of the two experimental factors (plant genetic group and soil compartment) and their interaction on α-diversity as characterized by the Shannon diversity index. Principal coordinate analyses were performed with the “ordinate” function in phyloseq using the Bray-Curtis distance function. Two way PERMANOVA was performed to analyze the impacts of the two experimental factors (plant genetic group and soil compartment) and their interaction on microbial community β-diversity. This analysis was performed using the “adonis” function in the vegan package in R with 999 permutations. Two additional R packages, DeSeq2 and indicspecies, were used to perform the differential abundance and indicator species analyses respectively^61,62^. The Benjamini-Hochberg procedure was used to adjust p-values to account for multiple comparisons. An adjusted p-value below 0.05 was considered significant.

### Network analysis of microbial co-occurrence/co-exclusion relationships

In order to construct a consensus network for each plant group, and remove potential habitat induced correlations caused by combining samples from different sample groups within the plant group, we used HabitatCorrectedNetwork, a previously described network construction method that incorporates corrections for both data compositionality and habitat filtering effects^63^. Separate microbial co-occurrence networks were constructed for each plant genetic group. Only plant accessions for which 3 replicates were successfully sequenced for all combinations of soil compartments (proximal and distal rhizosphere) and sequencing types (16S-V4 and ITS2) were included. Three replicates per subset of samples were necessary for the network construction algorithm used. Additionally, all networks were constructed from the same number of samples in order to avoid biases in network construction and statistical analysis that can arise from uneven sample sizes. Thus, each network was constructed from 12 samples (3 replicates of 2 accessions in the 2 soil compartments).

Networks were constructed separately for each plant group, applying the CLR transform to correct for data compositionality, and using the habitat correction algorithm to correct for effects of sample type (proximal vs. distal) and plant accession. After these corrections, Spearman correlations were used to construct correlation matrices using a significance cutoff of p < 0.05. Networks were analyzed with the NetworkX package in Python. Putative keystone taxa were identified in the co-occurrence networks based on their high weighted degree centrality (top 20% of ASVs in the network) and a low weighted betweenness centrality (<0.001, normalized)^34,64^.

The similarity of network co-occurrence patterns was determined based on the correlation matrices used to construct the networks. For the correlation matrix A, the value of A_ij_ is equal to the strength of correlation (ρ) between ASV_i_ and ASV_j_. Only ASVs detected in the rhizospheres of all three plant genetic groups were included in this comparison. This was necessary to allow direct comparison of the correlation matrices, in which the rows and columns must represent the same ASVs in each matrix being compared. The similarity measure used was the percent reduction (as compared to randomized resampling of the correlation matrices) in the matrix norm of the difference between the correlation matrices of the two networks being compared, as shown in Eq. (1).

1 $${\rm{Similarity}}=\frac{\Vert {{\rm{A}}}_{{\rm{rand}}}-{{\rm{B}}}_{{\rm{rand}}}\Vert -\Vert {\rm{A}}-{\rm{B}}\Vert }{\Vert {{\rm{A}}}_{{\rm{rand}}}-{{\rm{B}}}_{{\rm{rand}}}\Vert }\times 100$$

Here, A and B are the correlation matrices for the two networks being compared, and A_rand_ and B_rand_ are the randomly resampled matrices. A_rand_ and B_rand_ were estimated with 10,000 bootstraps, generating a distribution of similarity scores.

## Supplementary information

Supplementary Information

## Data Availability

The amplicon sequencing datasets generated during and analyzed during the current study are available through the JGI Genome Portal at https://genome.jgi.doe.gov/portal with the project ID 1138452.
