# Supplementary material for: Impacts of Maize Domestication and Breeding on Rhizosphere Microbial Community Recruitment from a Nutrient Depleted Agricultural Soil
Source: Sci Rep. 2019 Oct 30;9:15611. doi: 10.1038/s41598-019-52148-y (PMC6821752; doi:10.1038/s41598-019-52148-y)
Supplement: Supplementary file 1 — Supplementary Information [file 41598_2019_52148_MOESM1_ESM.pdf]

Impacts of Maize Domestication and Breeding on Rhizosphere Microbial Community Recruitment from a Nutrient Depleted Agricultural Soil

Supplementary Information

Vanessa Brisson, Jennifer E. Schmidt, Trent R. Northen, John P. Vogel, Amélie Gaudin

SUPPLEMENTARY FIGURES

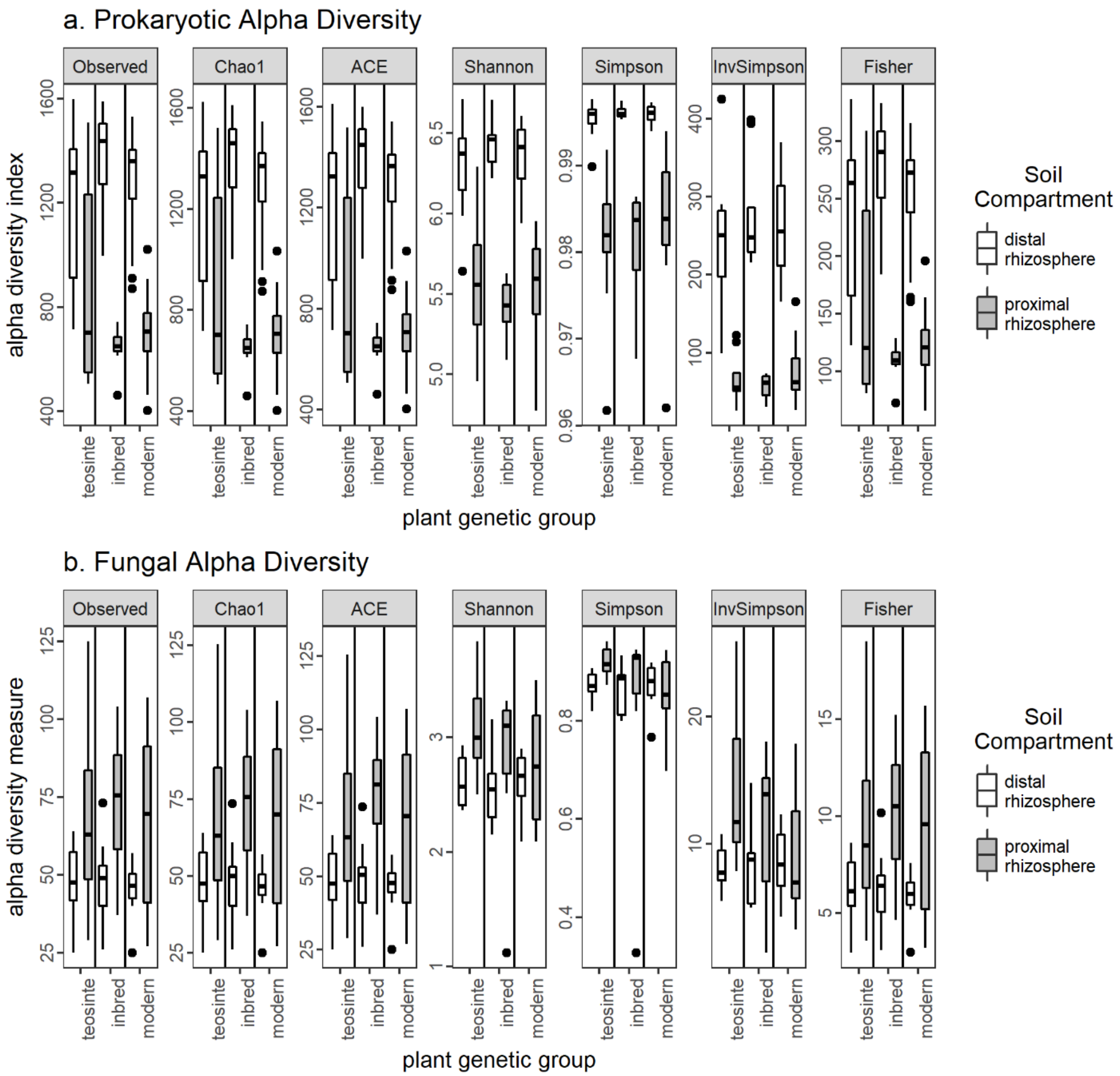

Supplementary Figure S1. Different measures of (a) prokaryotic, and (b) fungal α-diversity of distal (white) and proximal (grey) rhizosphere communities.

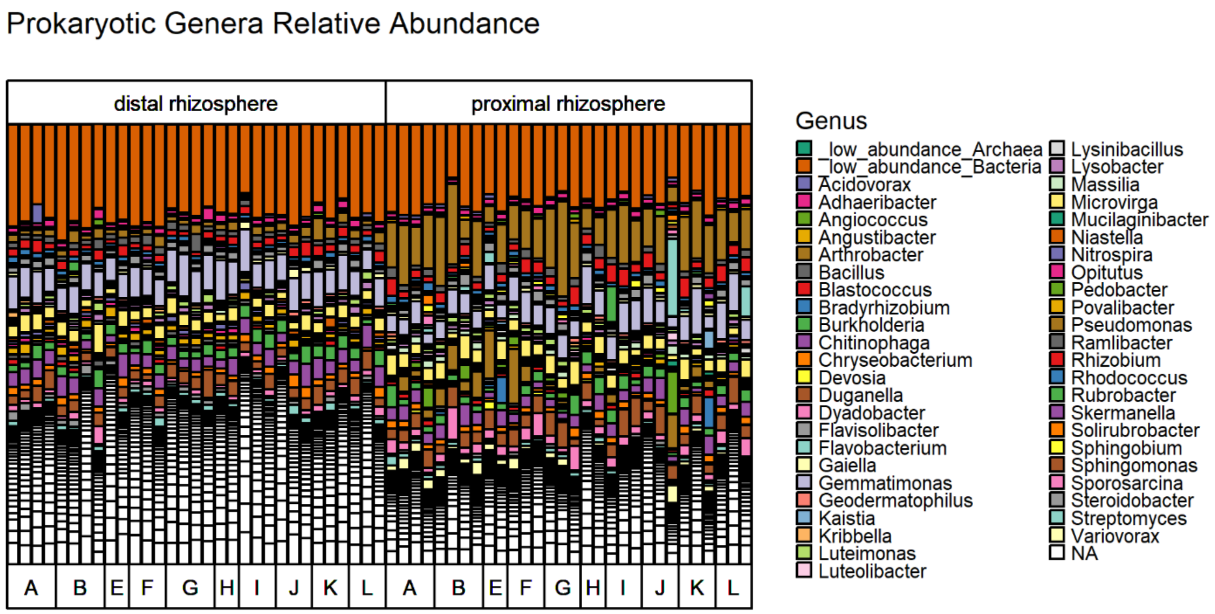

Supplementary Figure S2. Relative abundance of different taxonomic groups represented within the prokaryotic 16S-V4 sequences for individual rhizosphere samples grouped by soil compartment. Identifications are based on the RDP database.

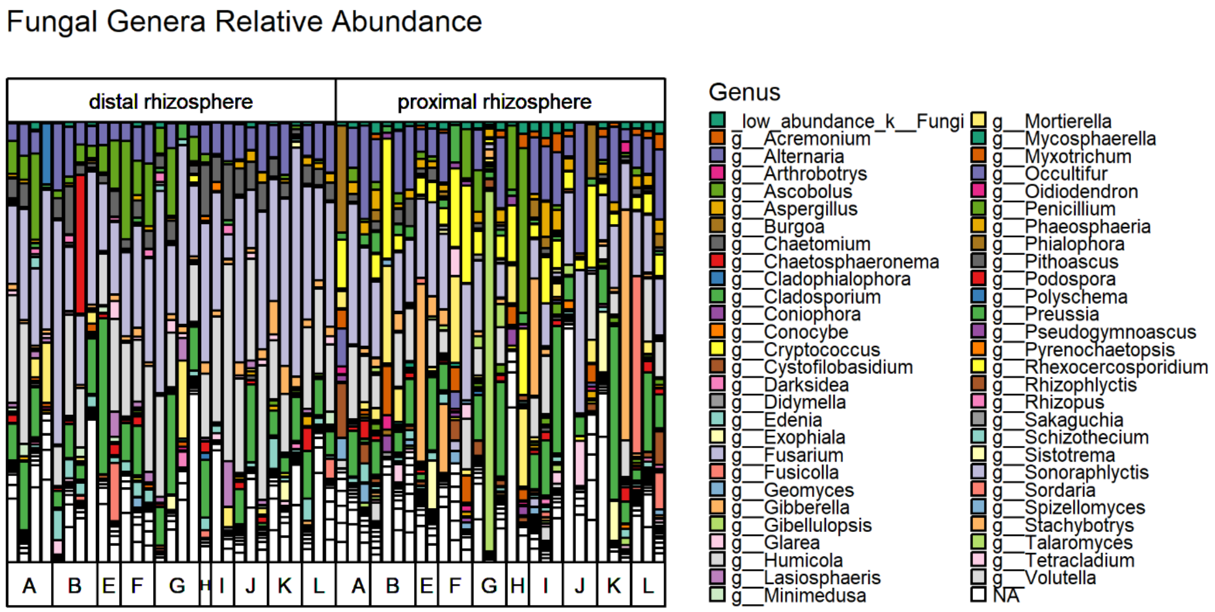

Supplementary Figure S3. Relative abundance of different taxonomic groups represented within the fungal ITS2 sequences for individual rhizosphere samples grouped by soil compartment. Identifications are based on the UNITE ITS database.

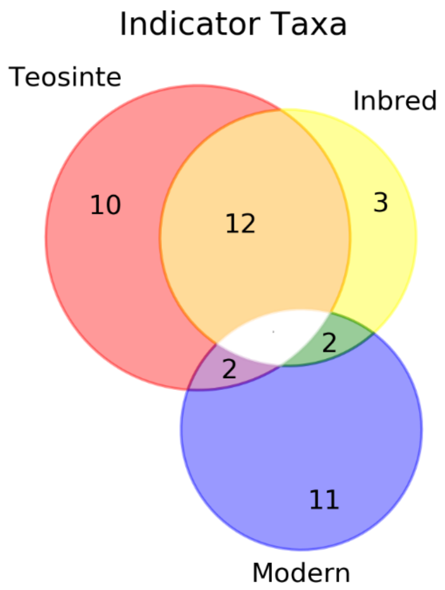

Supplementary Figure S4. Venn diagram of indicator taxa associated with individual plant genetic groups and with pairs of plant genetic groups.

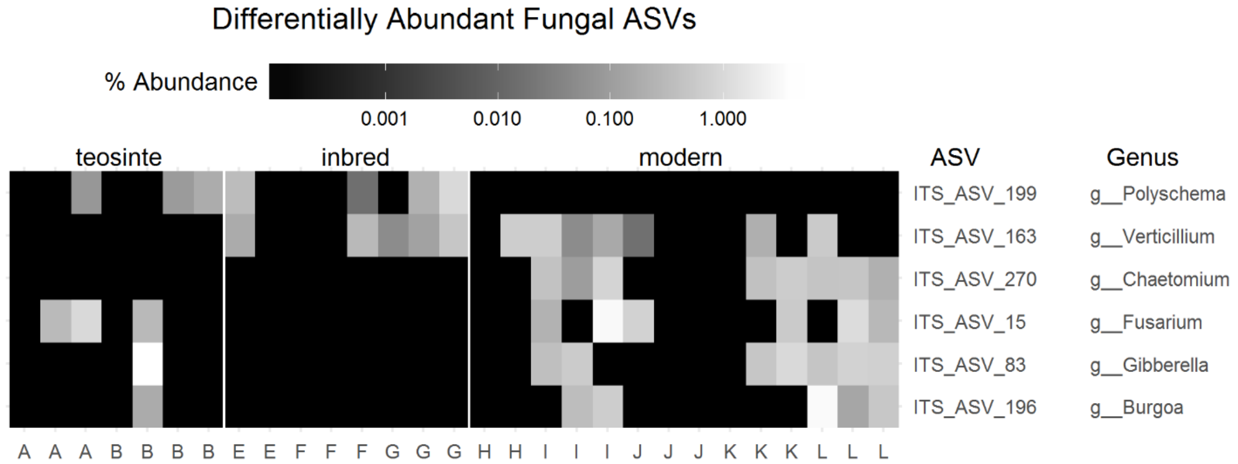

Supplementary Figure S5. Differentially abundant fungal ASVs. Rows represent individual ASVs. Columns represent individual samples. Samples are grouped by plant genetic group (top) and plant accession (bottom, letters correspond to plant accession IDs in Table 1). Brightness indicates relative abundance on a logarithmic scale.

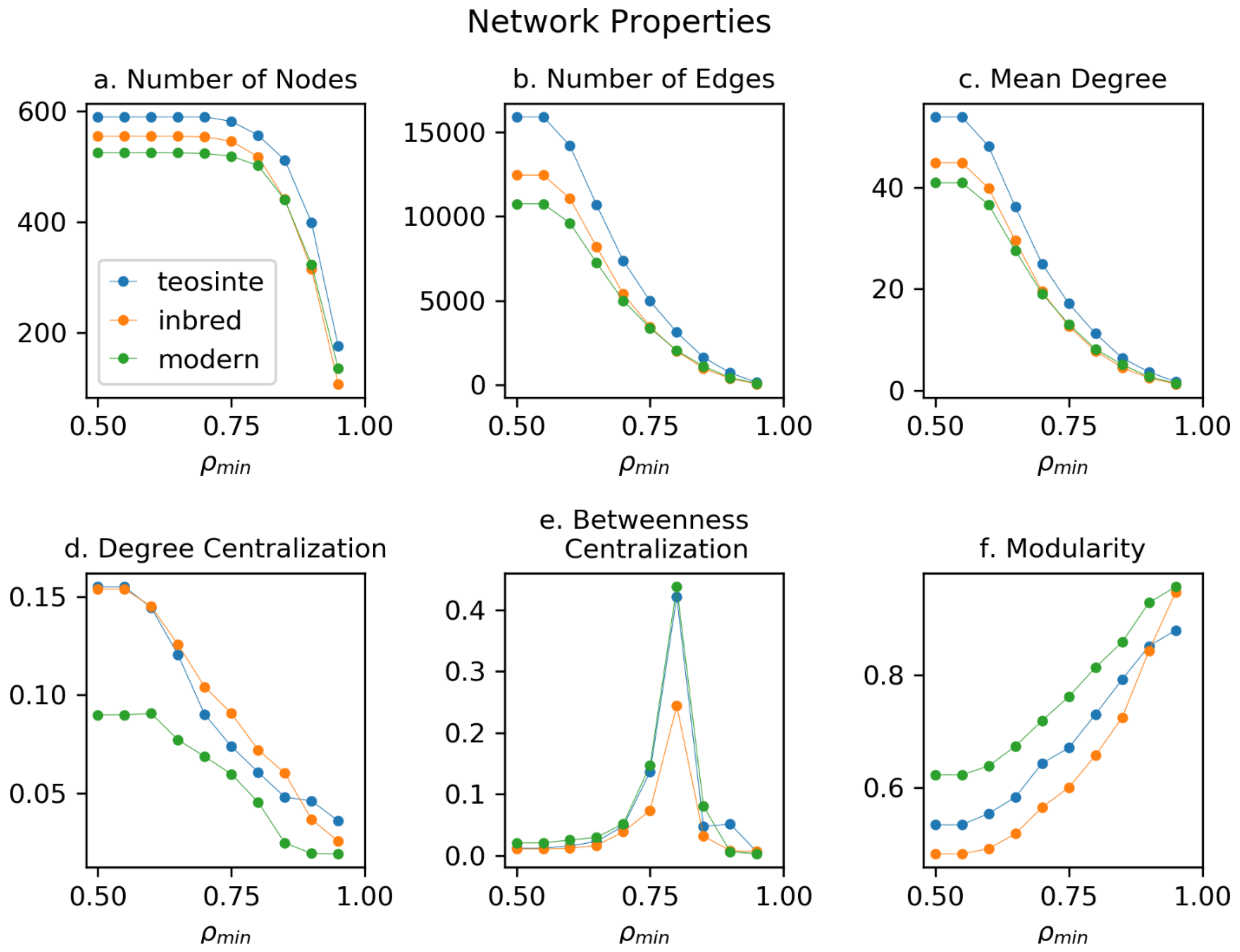

Supplementary Figure S6. Network properties of microbial co-occurrence networks constructed at different minimum correlation levels. (a) Number of nodes (ASVs with at least one signification correlation). (b) Number of edges (significant correlations between ASVs). (c) Mean degree (number of significant correlations per ASV). (d) Degree centralization. € Betweenness centralization. (f) Modularity.

SUPPLEMENTARY TABLES

Supplementary Table S1. ANOVA of prokaryotic α-diversity as represented by the Shannon diversity index. This is a two way type III ANOVA.

**Sources of Variation**	**Degrees of Freedom**	**Sum of Squares**	**F statistic**	**Pr(>F)**	**Statistical Significance**
soil compartment	1	10.4063	128.0732	5.52E-16	***
plant genetic group	2	0.0042	0.0258	0.9745	
soil compartment : plant genetic group	2	0.2549	1.5684	0.2176	

Significance codes: 0 ‘***’ 0.001 ‘**’ 0.01 ‘*’ 0.05 ‘.’ 0.1 ‘ ’ 1

Supplementary Table S2. ANOVA of fungal α-diversity as represented by the Shannon diversity index. This is a two way type III ANOVA.

**Sources of Variation**	**Degrees of Freedom**	**Sum of Squares**	**F statistic**	**Pr(>F)**	**Statistical Significance**
soil compartment	1	1.1538	5.9239	0.0184	*
plant genetic group	2	0.3254	0.8353	0.4395	
soil compartment : plant genetic group	2	0.2787	0.7155	0.4937	

Significance codes: 0 ‘***’ 0.001 ‘**’ 0.01 ‘*’ 0.05 ‘.’ 0.1 ‘ ’ 1

Supplementary Table S3. PERMANOVA of prokaryotic β-diversity as represented by Bray-Curtis distance metric. This is a two way PERMANOVA analysis with 999 permutations.

**Sources of Variation**	**Degrees of Freedom**	**Sums of Squares**	**Mean Squares**	**F statistic**	**Pr(> F)**	**Statistical Significance**
soil compartment	1	1.5889	1.58888	19.4321	0.001	***
plant genetic group	2	0.5250	0.26249	3.2103	0.002	**
soil compartment : plant genetic group	2	0.1843	0.09214	1.1269	0.262	
residuals	55	4.4971	0.08177			

Significance codes: 0 ‘***’ 0.001 ‘**’ 0.01 ‘*’ 0.05 ‘.’ 0.1 ‘ ’ 1

Supplementary Table S4. PERMANOVA of fungal β-diversity as represented by Bray-Curtis distance metric. This is a two way PERMANOVA analysis with 999 permutations.

Sources of Variation	Degrees of Freedom	Sums of Squares	Mean Squares	F statistic	Pr(> F)	Statistical Significance
soil compartment	1	1.6874	1.69744	7.7213	0.001	***
plant genetic group	2	0.7701	0.38506	1.7515	0.019	*
soil compartment : plant genetic group	2	0.4205	0.21025	0.9564	0.266	
residuals	52	11.4316	0.21984			

Significance codes: 0 ‘***’ 0.001 ‘**’ 0.01 ‘*’ 0.05 ‘.’ 0.1 ‘ ’ 1

Supplemental Table S5. Keystone Taxa. ASVs were identified as keystone taxa if they had a low weighted betweenness centrality (≤ 0.001) and a high weighted degree (top 20% within network). The ASV indicated in bold italic was identified as keystone for both the teosinte and modern networks. Other ASVs indicated in bold were identified as differentially abundant or indicator taxa. ASV identifications are based the RDP database. Identifications based on the Silva database are provided in parenthases wherever they differ from the RDP identifications.

Plant Group	ASV	Kingdom	Phylum	Genus	Mean Relative Abundance
teosinte	16S_ASV_8	Bacteria	Acidobacteria	NA (*RB41*)	0.44%
teosinte	***16S_ASV_13***	***Bacteria***	***α-Proteobacteria***	***Skermanella***	***0.67%***
teosinte	16S_ASV_17	Bacteria	Gemmatimonadetes	*Gemmatimonas* (NA)	0.43%
teosinte	16S_ASV_44	Bacteria	Gemmatimonadetes	*Gemmatimonas* (NA)	0.16%
teosinte	16S_ASV_53	Bacteria	Acidobacteria	NA	0.20%
teosinte	16S_ASV_66	Bacteria	NA (Tectomicrobia)	NA	0.16%
teosinte	16S_ASV_74	Bacteria	Actinobacteria	*Micromonospora* (NA)	0.25%
teosinte	16S_ASV_78	Bacteria	Acidobacteria	NA	0.16%
teosinte	16S_ASV_99	Bacteria	Acidobacteria	NA	0.20%
teosinte	16S_ASV_142	Bacteria	Acidobacteria	NA	0.28%
teosinte	16S_ASV_195	Bacteria	γ-Proteobacteria	NA (*Acidibacter*)	0.21%
teosinte	16S_ASV_199	Bacteria	β-Proteobacteria	*Burkholderia (Burkholderia-Paraburkholderia)*	0.31%
teosinte	16S_ASV_229	Bacteria	γ-Proteobacteria	*Luteimonas (Thermomonas)*	0.22%
teosinte	16S_ASV_302	Bacteria	Actinobacteria	*Solirubrobacter*	0.06%
teosinte	16S_ASV_303	Bacteria	Acidobacteria	NA	0.13%

teosinte	16S_ASV_342	Bacteria	Acidobacteria	NA (*Candidatus Koribacter*)	0.17%
**teosinte**	**16S_ASV_352**	**Bacteria**	**Gemmatimonadetes**	***Gemmatimonas***	**0.24%**
teosinte	16S_ASV_392	Bacteria	Acidobacteria	NA	0.12%
teosinte	16S_ASV_422	Bacteria	Acidobacteria (Chloroflexi)	NA	0.10%
teosinte	16S_ASV_423	Bacteria	β-Proteobacteria	NA	0.09%
teosinte	16S_ASV_472	Bacteria	Actinobacteria	*Solirubrobacter*	0.10%
**teosinte**	**16S_ASV_512**	**Bacteria**	**Actinobacteria**	***Pseudonocardia***	**0.07%**
teosinte	16S_ASV_621	Bacteria	Bacteroidetes	*Sediminibacterium* (NA)	0.08%
**teosinte**	**16S_ASV_637**	**Bacteria**	**Bacteroidetes**	***Flavisolibacter***	**0.10%**
teosinte	16S_ASV_1220	Bacteria	Actinobacteria	NA	0.01%
inbred	16S_ASV_2	Bacteria	Firmicutes	*Sporosarcina (Paenisporosarcina)*	1.93%
inbred	16S_ASV_3	Bacteria	α-Proteobacteria	*Microvirga*	2.95%
inbred	16S_ASV_26	Bacteria	Gemmatimonadetes	*Gemmatimonas* (NA)	0.61%
inbred	16S_ASV_71	Bacteria	Actinobacteria	*Rubrobacter*	0.23%
inbred	16S_ASV_100	Bacteria	Actinobacteria	*Streptomyces*	0.36%
inbred	16S_ASV_153	Bacteria	α-Proteobacteria	*Methylobacterium (Microvirga)*	0.07%
inbred	16S_ASV_227	Bacteria	Actinobacteria	*Solirubrobacter*	0.15%
inbred	16S_ASV_292	Bacteria	α-Proteobacteria	*Labrys*	0.07%
inbred	16S_ASV_412	Bacteria	Actinobacteria	*Gaiella*	0.04%
inbred	16S_ASV_1348	Bacteria	Actinobacteria	*Phycicoccus*	0.04%
modern	***16S_ASV_13***	***Bacteria***	***α-Proteobacteria***	**Skermanella**	***0.76%***
modern	16S_ASV_22	Bacteria	Actinobacteria	*Rubrobacter*	0.52%
modern	16S_ASV_64	Bacteria	α-Proteobacteria	*Anderseniella (Nordella)*	0.21%
**modern**	**16S_ASV_119**	**Bacteria**	**Actinobacteria**	***Solirubrobacter*** **(NA)**	**0.13%**
modern	16S_ASV_167	Bacteria	β-Proteobacteria	NA	0.40%
modern	16S_ASV_185	Bacteria	Bacteroidetes	*Flavisolibacter*	0.20%
modern	16S_ASV_216	Bacteria	β-Proteobacteria	NA	0.18%
modern	16S_ASV_226	Bacteria	Actinobacteria	*Dactylosporangium*	0.07%
modern	16S_ASV_234	Bacteria	β-Proteobacteria	NA	0.17%
modern	16S_ASV_270	Bacteria	α-Proteobacteria	*Microvirga*	0.09%
modern	16S_ASV_294	Bacteria	Acidobacteria	NA	0.11%
**modern**	**16S_ASV_415**	**Bacteria**	**Gemmatimonadetes**	***Gemmatimonas***	**0.28%**
modern	16S_ASV_430	Bacteria	α-Proteobacteria	*Sphingomonas*	0.16%
modern	16S_ASV_510	Bacteria	δ-Proteobacteria	NA (*Haliangium*)	0.11%
modern	16S_ASV_526	Bacteria	Acidobacteria	NA *(Bryobacter)*	0.07%

Supplementary Table S6. Soil management and properties. The first data column lists management practices and properties the soil used in this study. For comparison, the other columns show management and properties for soils collected from other plots (under different management practices) at the Russell Ranch Sustainable Agriculture Facility.

		**Nutrient Depleted (this study)**	**Conventionally managed**	**Organically managed**
**Management**	fertilization	unfertilized	synthetic fertilizer	compost and cover crop
	irrigation	rain-fed	drip irrigation	drip irrigation
	crop rotation	wheat/fallow	tomato/corn	tomato/corn
**Soil Properties**	Nitrogen (%)	0.096	0.098	0.175
	Carbon (%)	1.135	0.985	1.52
	Ammonia (ppm)	3.49	3.785	26.23
	**Nitrate (ppm)**	**2.425**	**30.755**	**38.78**
	**Phosphorus (Olsen) (ppm)**	**11.4**	**20.2**	**49.7**
	Potassium (ppm)	277	202.5	337
	Sodium (ppm)	10.5	41	132
	Calcium (meq / 100 g)	8.9	8.565	10.46
	Magnesium (meq / 100 g)	16.2	12.95	12.9
	Cation Exchange Capacity (meq / 100 g)	25.9	22.2	24.8
	Organic Matter (%)	3.02	2.57	3.5
	pH	6.695	7.015	7.41
	Sand (%)	22	35	32
	Silt (%)	46	38	40
	Clay (%)	32	27	28
